# Dynamics of carbon pools in post-agrogenic sandy soils of southern taiga of Russia

**DOI:** 10.1186/1750-0680-5-1

**Published:** 2010-04-26

**Authors:** Olga Kalinina, Sergey V Goryachkin, Nina A Karavaeva, Dmitriy I Lyuri, Luise Giani

**Affiliations:** 1 Dept. Soil Sci. C-v-O. University of Oldenburg, Oldenburg, Germany; 2 Institute of Geography, Russian Academy of Sciences, Moscow, Russia

## Abstract

**Background:**

Until recently, a lot of arable lands were abandoned in many countries of the world and, especially, in Russia, where about half a million square kilometers of arable lands were abandoned in 1961-2007. The soils at these fallows undergo a process of natural restoration (or self-restoration) that changes the balance of soil organic matter (SOM) supply and mineralization.

**Results:**

A soil chronosequence study, covering the ecosystems of 3, 20, 55, 100, and 170 years of self-restoration in southern taiga zone, shows that soil organic content of mineral horizons remains relatively stable during the self-restoration. This does not imply, however, that SOM pools remain steady. The C/N ratio of active SOM reached steady state after 55 years, and increased doubly (from 12.5 - 15.6 to 32.2-33.8). As to the C/N ratio of passive SOM, it has been continuously increasing (from 11.8-12.7 to 19.0-22.8) over the 170 years, and did not reach a steady condition.

**Conclusion:**

The results of the study imply that soil recovery at the abandoned arable sandy lands of taiga is incredibly slow process. Not only soil morphological features of a former ploughing remained detectable but also the balance of soil organic matter input and mineralization remained unsteady after 170 years of self-restoration.

## Background

Until recently, a lot of arable lands were abandoned in many countries of the world. Predominantly caused by economic crises, the most abandonment was found in Russia, reaching 578000 km^2 ^in the years 1961-2007 [1-3]. As a consequence, the soils of these sites underwent the process of natural restoration or self-restoration.

A recent chronosequential study on the succession of vegetation, profile morphology, and soil properties of post-agrogenic sandy soils under self-restoration of the southern taiga zone in the European part of Russia showed that the vegetation developed towards spruce forest and the soils towards natural Podzols with an accumulation of thick raw humus layers [4]. Additional podzolization features were found in respect to morphology and chemical properties like pH, exchangeable cations, and nutrition dynamics. Although these changes happened rather fast, the ploughing features were still evident after 170 years of self-restoration, as found in other studies [1,2,5,6].

Since every land-use change causes a disturbance of the long-termed adjusted balance of soil organic matter (SOM) supply and mineralization, self-restoration also leads to alterations in the SOM dynamics. In respect of afforestation of former arable sites, the most evident effect on C sequestration was the net sink of atmospheric CO_2 _with C accumulation mainly in the growing trees and the forest floor [7-11]. The mineral soil was found to account for less than 1% of radio carbon accretion [10] and might be even less due to varying interrelations between initial soil organic carbon (SOC) pools and SOC dynamics trends in respect to environmental conditions [12,13]. Results on C sequestration of post-agrogenic sandy soils under self-restoration of the southern taiga zone in the European part of Russia also indicate an over all sink [4]. Decreased SOC stores of the 0.2 m mineral soil were overcompensated by increasing SOC stores of the raw humus layer, the latter rating 10% of the whole SOC stores after 55 years of self-restoration, and 40% after 100 years and 52% after 170 years, respectively.

Self-restoration does not only affect carbon sequestration but also influences the different functional carbon pools and qualities. These changes in SOM are already indicated by increasing C/N ratios during self-restoration of post-agrogenic soils [4].

They are expected not to proceed consistently because they preferentially affect the SOM with a short turnover time corresponding to free particulate organic matter (OM) and the OM of the sand and coarse silt fractions [14-16]. Diminishing plant OM transformation induced an accumulation of particulate SOM from poorly decomposed plant residues producing raw humus in Podzols [11,17]. Because of low bioturbation, this SOM is considered for the SOM dynamics of the mineral soil. Therefore, during self-restoration, decreasing particulate SOM from former land use is expected in the mineral soil and perhaps simultaneously, increasing particulate SOM from fine root litter supplied by newly established plants. Changes in SOM dynamics are also expected for the mineral bound fraction. John et al. [16] reported more mineral bound organic carbon (OC) in cultivated soils than in forest topsoils. As discrete fractions show different times of decomposition in the range of 20-50 years for particulate SOM and about 100 years or more for mineral associated SOM [15,16,18], this decrease is assumed to be a long-term one at the site of this investigation.

To obtain additional information on C dynamics and alterations of soils under self-restoration, this study was carried out with a focus on different functional carbon pools and their qualities. We used the same soil chronosequence, which has been previously investigated for changes of basic soil properties [4].

## Results

The total SOC contents stayed relatively constant during self-restoration (Table 1). This was also obvious for the relictic ploughed horizons, showing a mean of 17.6 g kg^-1 ^for the 3 years old soil under self-restoration and 15.4 g kg^-1 ^for the 170 years old soil. Slight SOC enrichments were found in the newly developed Ah horizons 3-20 years after self-restoration with 23.7 and 29.9 g kg^-1 ^respectively, The SOC contents of the newly developed albic horizons were 36.1 and 21.7 after 100 and 170 years of self-restoration, whereas it was 20.9 in the same horizon of the Albic Podzol.

**Table 1:** Contents of total organic carbon and C/N ratios, free particulate organic matter (free POM) of the density fraction <1.8 g cm^-3^ and the mineral associated organic matter (<20 μm) of the density fraction >1.8 g cm^-3^ of the topsoils after 3, 20, 55, 100, and 170 years under self-restoration and of an Albic Podzol, never been cultivated.

Horizon	Depth	C	C/N	Free POM of the density fraction <1.8 g cm^-3^			Mineral associated OM (<20 μm) of the density fraction >1.8 g cm^-3^		
					
	cm	g kg^-1^		% of soil	% of SOC	C/N	% of soil	% of SOC	C/N
**3 years**									
Ah	10(12)^(1)^	23.7	15.6	1.33	56.2	12.5	0.62	26.3	12.1
	22	19.6	15.1	1.11	56.6	13.4	0.62	31.7	12.2
Ap1	33	19.1	16.5	0.81	42.6	12.7	0.48	25.2	12.7
Ap2	40	8.1	16.6	0.35	42.7	15.6	0.24	30.0	11.8
**20 years**									
Ah	2	29.9	14.6	2.25	75.3	14.9	0.61	20.3	13.7
Ap1	18	14.9	14.1	0.69	46.1	17.3	0.50	33.5	14.4
Ap2	25(28)	13.7	15.6	0.73	53.2	18.3	0.53	38.6	14.4
**55 years**									
Ah-E	1	nd	nd	nd	nd	nd	nd	nd	nd
Ap1	8	18.2	27.2	1.13	62.0	32.2	0.54	29.6	18.9
Ap2	19	12.3	27.0	0.68	55.1	33.8	0.39	32.0	18.8
**100 years**									
E	2	36.1	28.6	2.46	68.0	27.4	0.86	23.8	21.2
Bsh	5(9)	26.5	26.5	1.08	40.8	30.6	0.93	35.3	22.4
Ap	20	20.0	30.3	1.03	51.3	25.6	0.85	42.4	20.4
**170 years**									
E	2	21.7	31.2	1.53	70.5	27.9	0.54	24.9	19.0
Bsh	6	20.6	28.3	1.35	65.6	32.4	0.59	28.8	19.8
Ap	15	15.4	41.8	1.05	68.3	37.6	0.49	31.8	22.8
**Albic Podzol**									
E	6(8)	20.9	38.1	1.56	74.5	40.3	0.48	23.1	28.3
Bsh	10	26.4	35.7	1.03	38.9	35.7	0.85	32.2	26.5

nd - not determined

^(1) ^Depths descriptions in parentheses indicate partly thicker horizons

To test the occurrence of young living roots and to exclude the residual character from former land use Glomalin-related soil protein (GRSP) concentration was measured. As Glomalin is a protein produced by arbuscular mycorrhizal fungi during colonization, Glomalin concentration is correlated to the abundance of metabolizing roots [19]. GRSP concentration was 2.1 - 2.7 mg g^-1 ^in the former ploughed horizons of soils being 55, 100, and 170 years under self-restoration whereas it was 1.9 - 3.2 mg g^-1 ^in the Ah, Ap horizons of the 3 and 20 years' soils under self-restoration.

The OC content of free particulate organic matter (POM) in the light fraction (<1.8 g cm^-3^) was 38.9 - 75.3% of SOC (Table 1). The OC ratios of the particles' size <20 μm in the heavy fractions of the studied topsoils were in the range of 20.3 to 42.4% of SOC. Therefore, neither fraction showed a significant change during self-restoration. Occluded POM in the light fraction (<1.8 g cm^-3^) was not found. The C/N ratios of free POM in the light fraction (<1.8 g cm^-3^) increased under self restoration: 13.8 - 16.8 - 33.0 - 27.9 - 32.6 (in the mean) after 3, 20, 55, 100, and 170 years of abandonment. The C/N ratio of the free POM in the light fraction (<1.8 g cm^-3^) of the Albic Podzol was with 36 in the mean remarkably high. The C/N ratios of OM of the particles size <20 μm in the heavy fraction (>1.8 g cm^-3^) also increased within self-restoration: 12.2 - 14.2 - 18.9 - 21.3 - 20.5 (in the mean) after 3, 20, 55, 100, and 170 years of abandonment. The Albic Podzol showed a C/N ratio 27.4. IR spectroscopic analysis of the OM in the light fractions did not show differences within self-restoration in respect to the composition of the functional groups as well as to their relative rates (data not shown).

The OC contents in the sand and coarse silt size separates was 47.4 - 73.9% of SOC (Table 2). The OC contents in the fine silt and clay fraction were in a range of 12.5 to 34.4% of SOC. As a result, both fractions did not show a significant change during self-restoration. The N contents in the sand size fractions of ploughing horizons were under detecting limit from 3 years of self-restoration and thereafter (Table 3). The C/N ratios of OM of the fine-silt and clay fraction also increased within self-restoration but with less extent, showing C/N ratios of 12 (mean values) at the beginning and 20 at the end of the chronosequntial observation for the silt and 11 and 15 for the clay fraction, respectively.

**Table 2:** Contents of organic carbon within the grain size fractions of the topsoils after 3, 20, 55, 100, and 170 years under self-restoration and of an Albic Podzol, never been cultivated.

		OC content in grain size					
		
		Sand		Silt			Clay
			
Horizon	Depth	2.0 - 0.2 mm	0.2 - 0.063 mm	0.063 - 0.020 mm	0.020 - 0.0063 mm	0.0063 - 0.002 mm	<0.002 mm
		
	cm	% of SOC					
**3 years**							
Ah	10(12)^(1)^	33.0	19.3	21.6	8.4	10.8	6.3
Ap1	22	34.6	15.8	22.2	5.7	13.9	7.5
	33	35.0	9.5	16.4	6.9	10.8	8.7
Ap2	40	39.1	6.5	11.1	8.2	14.1	13.1
**20 years**							
Ah	2	38.9	12.3	20.5	8.6	10.4	8.3
Ap1	18	20.4	10.5	17.1	12.5	16.6	15.2
Ap2	25(28)	22.5	9.4	21.2	12.2	21.3	13.1
**55 years**							
Ah-E	1	Nd	nd	nd	nd	nd	nd
Ap1	8	32.2	6.8	10.1	12.3	11.4	9.1
Ap2	19	24.4	7.8	15.2	21.7	13.6	13.3
**100 years**							
E	2	43.1	3.3	23.9	7.2	13.1	9.1
Bsh	5	41.3	5.1	15.2	15.3	10.9	6.8
Ap	10	37.1	6.5	16.4	23.6	8.9	7.0
**170 years**							
E	2	50.1	4.4	14.5	9.0	13.8	8.1
Bsh	6	31.2	4.9	16.2	12.7	14.1	9.3
Ap	15	30.5	9.1	17.3	16.4	10.1	13.8
**Albic Podzol**							
E	6(8)	41.6	5.2	16.7	13.3	12.6	10.4
Bsh	10	35.4	7.3	8.8	6.3	6.0	6.5

nd - not determined

^(1) ^Depths descriptions in parentheses indicate partly thicker horizons

**Table 3:** C/N ratios of the organic matter within the grain size fractions of the topsoils after 3, 20, 55, 100, and 170 years under self-restoration and of an Albic Podzol, never been cultivated.

		C/N ratios in grain size					
		
		Sand		Silt			Clay
				
Horizon	Depth cm	Coarse/Medium	Fine	Coarse	Medium	Fine	
				
		2.0-0.2	0.2-0.063	0.063-0.020	0.020-0.0063	0.0063-0.002	<0.002
		
		mm					
**3 years**							
Ah	10(12)^(1)^	31.4	28.8	14.0	12.9	12.0	10.7
Ap1	22	25.0	24.2	14.2	12.1	12.0	11.2
	33	34.7	nd	14.9	14.3	12.0	10.9
Ap2	40	nd	nd	23.2	14.9	12.0	9.6
**20 years**							
Ah	2	21.0	24.5	16.5	13.2	12.4	8.3
Ap1	18	nd	nd	19.0	15.5	13.5	9.2
Ap2	25(28)	nd	nd	21.3	18.0	15.5	10.1
**55 years**							
Ah-E	1	nd	nd	nd	nd	Nd	nd
Ap1	8	nd	nd	38.9	24.2	23.8	13.2
Ap2	19	nd	nd	45.2	26.3	24.2	15.0
**100 years**							
E	2	nd	nd	29.8	30.4	21.7	18.9
Bsh	5	nd	nd	32.0	26.7	21.5	15.1
Ap	10	36.7	nd	30.9	24.0	21.6	13.7
**170 years**							
E	2	53.1	42.8	24.0	24.4	18.5	14.8
Bsh	6	nd	nd	30.5	28.6	17.1	14.6
Ap	15	nd	nd	46.9	29.9	24.9	17.5
**Albic Podzol**							
E	6(8)	nd	nd	44.6	38.0	28.5	17.7
Bsh	10	nd	nd	31.6	26.8	22.9	17.5

nd - not determined

^(1) ^Depths descriptions in parentheses indicate partly thicker horizons

## Discussion

This chronosequence showed an enrichment in SOC during self-restoration [4], which was caused by the development of an organic surface horizon and not by changes within the mineral horizons (Table 1). In the mineral horizons only slight modifications and peculiarities were observed, including enrichment in the newly developed Ah horizons 3-20 years after self-restoration, relatively high C contents within the newly developed albic horizons compared to natural Podzols [20], and relatively constant C contents within relict ploughed horizons. The latter confirms the morphological findings of long existing Ap features and corresponds to the high phosphorus contents even after an abandonment time of 170 years [4] and high root densities. Consequently, the former ploughed horizons act as a favoured rooting zone for the newly established vegetation. This assumption was confirmed by the occurrence of high Glomalin-related soil protein (GRSP) concentrations. Generally, the SOC contents of the mineral horizons did not change during self-restoration, indicating quantitatively balanced carbon dynamics during this process.

Occluded POM in the light fraction (<1.8 g cm^-3^) was not found, confirming the morphological findings of no aggregates in the studied soils.

With larger amounts in the top than in the subsoils, the highest OC content was found for free POM in the light fraction (<1.8 g cm^-3^) and in the sand and coarse silt size separates (Table 1, 2). Composed of macroorganic matter from plant and animal origin [14], this SOC refers to the active and intermediate pool if charcoal is negligible with a short turnover time from 1 to 100 years [18]. Although a lower amount of active and intermediate OC pools in the arable soil than in the forest soil have been documented in several studies [7,16,21-23], we did neither find increasing SOC in the free POM of the light fraction (<1.8 g cm^-3^) nor in the sand and coarse silt size separates with increasing duration of self-restoration (Table 1, 2). As previously stated, we assume that the temporal constant amounts of these fractions in the mineral topsoil during self-restoration were caused by compensation of the loss of these fractions from the agronomic phase via mineralization and subsequent humification of newly developing roots. These fractions show an adjusted carbon balance although mineralization and carbon sequestration occurred, confirming other studies [24-26] which state, that the light fraction is not an universal indicator in relation to land use change.

The OC of the heavy fraction (>1.6-2.0 g cm^-3^) and fine-silt and clay fraction includes organo-clay complexes and mineral grains coated with OM, representing a stable SOC or passive pool [14,18] with turnover times of 10 - 100 years and more for OC in the fine-silt and definitely more than 100 years in the clay fraction. Consequently, OC of the particles size <20 μm released from density fraction >1.8 g cm^-3 ^and the OC of the fine silt and clay fraction, investigated in this study, compose a stable OC pool with long turnover times. According to John et al. [16], a decrease of this pool was expected for the studied chronosequence. Nevertheless, the OC ratios of the particles' size <20 μm in the heavy fractions of the studied topsoils were in the similar range to the OC contents in the fine silt and clay fraction (Table 1, 2), showing no quantitative alterations of the stable OC pools with increasing duration of self-restoration which was unexpected because of increasing podzolisation dynamics but indicates once again an adjusted carbon balance.

The C/N ratios of free POM in the light fraction (<1.8 g cm^-3^) increased within self restoration (Table 1). These C/N ratios were expected to reach the value of the Albic Podzol (Table 1) but because of particular charcoal enrichment in the latter [4] they did not. The increase of C/N ratios in these fractions resulted from increasing mineralization of agriculture-derived SOM and from the gradual input of qualitatively different organic material with low nitrogen concentration as discussed above. The nitrogen being released in mineral topsoil due to SOM mineralization is assimilated by the vegetation, creating a loss for the mineral soil [17]. As plant available phosphorus and potassium showed the same behaviour within this chronological sequence [4] an over-all shifting of the source of plant nutrients upwards to the forest floor was observed and documented in respect of principle raw humus formation processes by Chertov [27] and Chertov et al. [28].

Although no quantitative modifications of total SOM was observed, the changing C/N ratios of free POM in the light fraction (<1.8 g cm^-3^) reflected qualitative alterations during self-restoration. This process could not be seen via IR spectroscopic analysis (data not shown). Highest and no more increasing C/N values, achieved after 55 years of self-restoration (Table 1), indicated a new qualitative OC balance being present after that time. The N content in the sand size fractions of ploughing horizons was under detecting limit (Table 3), indicating partial rotation of an active OC pool already after 20 years of self-restoration. Short rotation times were also found for other environmental conditions [16,29]. For this study, the quick SOM rotation is considered to be due to "fresh" and easy decomposable macroorganic residues being characteristic of these OC fractions [14] in combination with accelerated acidification within this chronosequence [4], the latter also discussed by Chertov and Menshikova [30].

Qualitative changes in SOM within self-restoration were also found by increasing C/N ratios for OM of the particles' size <20 μm in the heavy fraction (>1.8 g cm^-3^) (Table 1) and for OM of the fine-silt and clay fraction (Table 3). Lower C/N ratios with decreasing grain sizes indicate less changes of SOM in the same order. These results confirm that SOM changes also affect the stable OC pool, also reported by others [16,23,29,31], showing mean ages of stable SOC in the range from 50 to 260 years. According to the C/N ratios in the heavy fraction and fine-silt clay fractions, the first clear qualitative alterations of the stable OC pool found in this study occurred after 55 years of self-restoration. This alteration might be due to portions of rapid cycling C, being amongst the heavy fraction with long turnover times according to Golchin at al. and Swanston et al. [32-34], in combination with quick podzolisation found for this chronosequence [4], as already discussed above. Supply might be induced by highly decomposed OM from root litter and by leaching of highly oxidized water-soluble organic products from the forest floor into the mineral soil horizons and stabilisation therein by association with mineral phases [35]. Although the initiation was quick, "a complete new qualitative balance" of the stable SOM was not achieved within 170 years of self-restoration. This is shown by the C/N ratios of OM of the particles' size <20 μm in the heavy fraction (>1.8 g cm^-3^) and in the fine-silt and clay fractions at the end of the chronosequence (Table 1, 3), being lower than those of the Albic Podzol.

## Conclusion

Although the sandy post-agrogenic soils in the southern Taiga sub-zone of European Russia indicated a carbon sink during self-restoration by increasing carbon sequestration within the developing organic surface layers, the mineral horizons showed only small alterations in total OC contents and no quantitative but qualitative modifications in respect of different functionally OC pools. The constant quantities are supposed to result from simultaneously running supply and mineralization processes. The changing qualities indicate the formation of "a new balance" of active SOM within some decades, whereas this process is not yet completed for the passive SOM after 170 years of self-restoration. Also expressed by remaining soil morphological ploughing features, this study confirms that carbon dynamics are not balanced after 170 years of self-restoration. Soil recovery from agriculture is a long-term or infinite process.

## Methods

### Site of investigation

The study was done at the same sites where Kalinina et al. [4] conducted their investigation. It was located in the southern boreal sub-zone of the European part of Russia nearly 12-30 km south of the town Valday. Valday is situated at the federal highway M10, connecting Moscow with St. Petersburg, both being roughly 400 km apart.

Geographically, the investigation site is a part of the Valday Hills on the east European plain. The average annual temperature is +3.2°C, the annual precipitation is 714 mm, and the frost-free period covers 128 days [36]. The Valday Hills were formed by late Weichselian end moraines, consisting of hills and many lakes in the depressions. Loamy to clayey as well as sandy sediments constitute the soil development. The latter were chosen for this study. For the chronosequential approach of this study, sites different in self-restoration time but comparable in soil texture, climate, and land-use history were selected. Subsequent sampling sites were chosen from information obtained from topographic and geological maps, historical literature, and personal communication with local people. Having found adequate sites, five post-agrogenic Podzols profiles of different self-restoration ages were dug in September 2007. Frequent Purckhauer drilling ensured that all profiles were representative of the sites. The chronosequential catena covered 3, 20, 55, 100, and 170 years of self-restoration. For precise locations and photos of the soil profiles see Kalinina et al. [4]. The sand percentage of all soils was >85% and the clay fraction ≤2%, indicating their pedological similarity. An Albic Podzol, which had never been under agricultural use, was included as a control. Soil morphology was described according to the Russian taxonomy [37] and World Reference Base of Soil Resources (WRB) [38]. Bulk and volume samples were taken in triplicate from every horizon; the former ploughed horizons were additionally sampled at 10 cm intervals with increasing depths.

### Glomalin-related soil protein extraction

Glomalin-related soil protein (GRSP) was extracted following the method of Wright and Upadhyaya [39], modified by Halvorson & Gonzales [40]. Briefly, 2 g of soil were extracted by a total volume of 50 ml 50 mM diphosphate (adjusted to pH 8.0) at 128°C during four extraction cycles, each lasting 1 hour. The pooled extract was centrifuged to remove soil particles and protein concentration determined by the Bradford assay [41] using BSA (bovine serum albumin) as a standard.

### Density and particle size fractionation

The procedure was conducted in duplicate to obtain free particulate organic matter (POM) and occluded POM of the light fraction (<1.8 g cm^-3^) and fraction of particles <20 μm of the heavy fraction (>1.8 g cm^-3^) [42]. To avoid slaking, a 30 g air-dried soil sample <2 mm was capillary-saturated and then immersed in deionised water on a 20 μm mesh sieve for 5 min. The sieve was gently stirred on a rotating shaker (Laborshake Gerhardt Bonn) for 100 revolutions at a frequency of 55 rpm, dried at 40°C, weighed, and the soil material was transferred to a centrifuge tube together with sodium polytungstate at a density of 1.8 g cm^-3 ^(TC-Tungsten Compounds, Germany). The filling level was 2/3 of the tube. The tube was hand-shaken ten times, and then centrifuged at 1200 g for 10 min. The supernatant was washed with deionised water onto a paper filter (weight band Rotilabo) until the electrical conductivity dropped to <50 μS cm^-1^. The remaining solution was filtered through a Glass Microfibre filter (GF/A) (referred to as free POM of the light fraction (<1.8 g cm^-3^)). The residual soil material was dispersed ultrasonically (HF-Generator GM-2200, Sonotrode VS 70T, Bandelin) with energy of 450 J ml^-1 ^for complete dispersion of soil micro aggregates [43]. The instrument was calibrated calorimetrically every 4 weeks according to North [44]. The dispersed soil was re-sieved at 20 μm. The material on the sieve was dried and treated as above to separate the occluded POM, which was not found. The fine fraction <20 μm was obtained by shaking a horizontal shaker at the beginning of the procedure and the fine fraction <20 μm gained after re-sieving the ultrasonically dispersed soil was combined and obtained by centrifugation by 4000 g for 18 min. The supernatant was removed and the settled sample was washed intensively with deionised H_2_O. The procedure was repeated until the electrical conductivity was <50 μS cm^-1 ^and the supernatant was clear. Thereafter, the sample was dried at 40°C and weighed (referred to mineral-associated OC (<20 μm) of the heavy fraction (>1.8 g cm^-3^). All fractions were analyzed for total C and N.

The free POM of the light fraction was additionally analyzed by infrared spectroscopy (FTS 7, Fa. Bio-Rad). Duplicate samples of 3-4 mg were ground with 100 mg KBr and pressed (8 t) to a pellet. The subsequent measurements were carried out in the mid-infrared area (225 - 4000 cm^-1^). The spectra were transformed into their real OC quantities of total C. The interpretation of the IR spectra was done according to Stevenson [45], Fookeu [46] and Senesi et al. [47].

### Grain-size fractionation

Grain-size fractionation was done according to Kaiser et al. [48]. Destruction of aggregates and sample dispersion was achieved by a two-step ultrasonication (HF-Generator GM-2200, Sonotrode VS 70T, Bandelin). The instrument was calibrated calorimetrically every 4 weeks according to North [44]. The energy used for separating sand particles >200 μm was 60 J ml^-1^. The energy input for the second dispersion step was 440 J ml^-1^. Sand and coarse silt particles (>20 μm) were separated by wet-sieving. Medium silt (20-6.3 μm) and fine silt (6.3-2 μm) fractions were recovered by sedimentation. The clay-size particles (<2 μm) were separated by centrifugation at 4400 g for 20 min. Then, the particle-size separates were dried at 105°C and analyzed for total C and N.

The sum of the fractions is lower than 100% because of the specification of the fractionation technique: OC measurement in many fractions, OC loses, due to sodium polytungstate, application [32,49-52].

Carbon and nitrogen contents in dry soil pellets were determined after combustion and spectrometric measurements with a C/N/S analyser (CHNS-Analyser Flash EA) within the density and particle size fractions <20 μm as well as within the grain-size fractions (sand, silt, clay).

Unless stated otherwise, the data were based on three replicates; the standard deviations were always less than 10% of the mean.

## Competing interests

The authors declare that they have no competing interests.

## Authors' contributions

OK carried out literature research, participated in soil sampling, performed soil analysis, and drafted the manuscript. SVG and DIL conceived of the project idea, carried out the study of historical land use, participated in soil sampling. NAK helped in characterisation of the soils. LG is participated in coordination of the study and its design, and drafted the manuscript. All authors read and approved the final manuscript.
